# Clinical experience with ventriculovesical shunting as a salvage or bridging strategy in children with hydrocephalus when peritoneal diversion was considered unsuitable: a retrospective case series

**DOI:** 10.3389/fped.2026.1923657

**Published:** 2026-10-13

**Authors:** Lixue Shen, Yangxu Gao, Yu Li, Shulei Wang, Hongxin Yao

**Affiliations:** Department of Pediatric Surgery, Peking University First Hospital, Beijing, China

**Keywords:** cerebrospinal fluid diversion, pediatric hydrocephalus, salvage shunt, urinary bladder, ventriculovesical shunt

## Abstract

**Objective:**

Ventriculoperitoneal (VP) shunting remains a standard cerebrospinal fluid (CSF) diversion procedure for pediatric hydrocephalus. However, peritoneal diversion may be considered unsuitable in selected children because of abdominal infection, severe peritoneal pathology, refractory CSF ascites, or persistent CSF abnormalities. The objective of this study was to describe our institutional experience with ventriculovesical shunting (VVS) as a selective salvage or bridging CSF diversion strategy in such patients.

**Materials and methods:**

We retrospectively reviewed 36 children with hydrocephalus who underwent VVS at our center between September 2014 and January 2022. The decision to use VVS was individualized according to the clinical circumstances and the treating team's assessment that peritoneal diversion was undesirable or not feasible at that time. Available reasons to avoid peritoneal diversion, postoperative complications and conversion to subsequent VP shunting were reviewed.

**Results:**

All 36 VVS procedures were technically completed. Follow-up ranged from 6 to 24 months (mean, 13.44 ± 8.21 months). At the most recent available follow-up, radiological improvement of hydrocephalus was reported in 27 patients, while seven remained clinically stable; two patients discontinued treatment and were lost to follow-up. Five patients (13.9%) developed postoperative intracranial infection, including four (11.1%) within the first postoperative month. Three patients (8.3%) developed symptomatic hyponatremia requiring sodium supplementation. Eleven patients (30.6%) were subsequently converted to VP shunting when their clinical circumstances permitted. Urological safety outcomes were incompletely ascertained.

**Conclusions:**

In this retrospective single-center series, VVS provided internal CSF diversion during the available follow-up period in a highly selected group of children for whom peritoneal diversion was considered unsuitable at the time of treatment. These observations support describing VVS as a potential salvage or bridging strategy. Prospective studies incorporating longer follow-up are required before broader conclusions regarding patient selection and safety can be made.

## Introduction

Hydrocephalus is a common condition in pediatric neurosurgery, and ventriculoperitoneal (VP) shunting remains the most widely used CSF diversion procedures. Nevertheless, the peritoneal cavity may become an unfavorable recipient site in selected patients, particularly in the presence of active or recent abdominal infection, severe adhesions or loculated abdominal collections, and refractory ascite s (1, 2). When peritoneal diversion cannot be used, alternative strategies include external ventricular drainage (EVD), endoscopic third ventriculostomy (ETV) when anatomically appropriate, ventriculoatrial (VA) shunting, and ventriculopleural (VPl) shuntin g (3–7). Selection among these options is individualized according to hydrocephalus etiology and anatomy, patient age, infectious status, previous procedures, and the risks associated with the proposed distal recipient site.

Venticulovesical shunting is an uncommon alternative in which CSF is diverted into the urinary bladder. Published clinical experience with this technique remains limited, and contemporary pediatric data continue to characterize it predominantly as a salvage procedure rather than a routine second-line diversion route (8). In particular, long-term effects of chronic exposure of the urinary tract to CSF and an indwelling catheter remain incompletely defined.

Persistently elevated CSF protein was one of the clinical factors that led our team to avoid immediate peritoneal diversion in some patients during the study period. However, the relationship between CSF protein concentration and shunt failure remains inconsistent in the literature (9). Accordingly, elevated CSF protein should not be regarded as a universally established contraindication to VP shunting, and no universally accepted numerical threshold for avoiding VP shunting has been established.

The present study therefore does not aim to establish specific indications for VVS or to demonstrate superiority over other CSF diversion strategies. Instead, we retrospectively describe our institutional patient-selection pathway, perioperative management, short- to intermediate-term clinical outcomes and infectious complications follow-up in 36 children who underwent VVS when conventional peritoneal diversion was considered undesirable or not feasible.

## 1 Materials and methods

### 1.1 Materials

#### 1.1.1 General information

Between September 2014 and January 2022, 36 children with hydrocephalus underwent VVS after the treating team considered peritoneal diversion undesirable or not feasible at that point in their clinical course. The cohort comprised 21 boys and 15 girls, with ages ranging from 3 months to 11 years (mean, 35.7 ± 33.6 months). All patients met the diagnostic criteria for hydrocephalus, including ventricular enlargement and/or elevated intracranial pressure accompanied by neurological manifestations such as vomiting, irritability, or developmental delay. Diagnosis was established through clinical evaluation and neuroimaging, including computed tomography (CT) or magnetic resonance imaging (MRI).

All patients underwent preoperative assessment, including routine laboratory testing, renal function, serum electrolytes, urinalysis, and urine culture. Informed consent was obtained from the families of all patients, and the study was conducted in accordance with institutional ethical standards.

#### 1.1.2 Clinical factors leading the treating team to avoid peritoneal diversion

The decision not to perform VP shunting at the time of VVS placement was based on an individualized clinical assessment rather than on a set of universally accepted absolute contraindications. Four principal clinical scenarios were identified in the retrospective records.

**Persistent CSF abnormality after bacterial meningitis**: Persistent post-infectious CSF abnormalities after bacterial meningitis: Seventeen patients (47.2%) had persistent CSF abnormalities following treatment for bacterial meningitis. Repeated CSF cultures were negative after targeted antimicrobial therapy, whereas CSF protein remained above the laboratory reference range for more than 1 month, ranging from 1.02 to 11.25 g/L (mean, 4.47 ± 3.68 g/L). No predefined or validated CSF protein threshold was used to select patients for VVS, and elevated CSF protein was not regarded as an absolute contraindication to VP shunting.

Abdominal infections: Abdominal infection or chronic inflammatory abdominal changes were present in 12 patients (33.3%). Imaging findings included gas-fluid levels or encapsulated fluid collections. Nine of these patients had developed abdominal infection after previous VP shunting.

Post-fungal meningitis sequelae: Three patients (8.3%) had persistent CSF abnormalities following fungal meningitis. Despite antifungal treatment and repeated negative serum and CSF fungal cultures, abnormalities in CSF white blood cell count, protein, and glucose persisted for more than 1 month (10).

Recurrent massive abdominal ascites: Four patients (11.1%) had recurrent massive abdominal ascites after VP shunting. Other clinically recognized causes such as hypoproteinemia were excluded, and ascites persisted despite repeated abdominal puncture or external drainage.

#### 1.1.3 Patient-selection pathway

VVS was considered only after review of the patient's previous CSF diversion history, current infectious status, abdominal conditions, and feasibility of other diversion strategies. Available information regarding previous VP shunting and other CSF diversion procedures was retrospectively extracted from the medical records and is summarized in Table 1. At our center, none of the patients included in the present study had a history of treatment with VA, VPl, or ETV.

**Table 1:** Previous CSF diversion history and clinical pathway leading to ventriculovesical shunting.

Clinical scenario	n	Reasons why peritoneal diversion was avoided at VVS placement	Documented previous VP shunt	Documented previous EVD	Documented previous Ommaya reservoir placement	Why VVS was selected
Persistent CSF abnormality after bacterial meningitis	17	Persistent CSF abnormality after cultures became negative; institutional concern regarding immediate peritoneal diversion	0	14	3	Individualized institutional salvage/bridging decision
Abdominal infection/chronic inflammatory change	12	Abdominal infection/inflammatory environment unsuitable for distal peritoneal placement	9	5^a^	0	Avoidance of diseased peritoneal recipient site
Persistent abnormalities after fungal meningitis	3	Persistent CSF abnormalities after microbiological control	0	1	3	Individualized nonperitoneal diversion
Refractory CSF ascites	4	Recurrent ascites despite puncture/external drainage	4	0	0	Alternative recipient site while peritoneum was ineffective
Total	36	—	13	20	6	—

a Previous procedures were not mutually exclusive; individual patients may have undergone more than one temporary CSF diversion procedure.

### 1.2 Treatment methods

#### 1.2.1 Treatment plan

All 36 patients underwent ventriculovesical shunting. Programmable valves were used in 27 patients, with an initial setting of 14 cmH_2_O, whereas nine patients received fixed-pressure valves rated at 11 cmH_2_O. No symptoms suggestive of pre-existing urinary tract disease were documented before surgery, and preoperative urinalysis and urine culture were unremarkable in all patients. However, systematic preoperative urodynamic testing or imaging specifically designed to detect vesicoureteral reflux was not part of the study protocol.

#### 1.2.2 Surgical Procedure

All procedures were performed under general anesthesia. The ventricular catheter was inserted using standard neurosurgical techniques. The distal catheter was then implanted into the bladder with the assistance of pediatric urologists. A suprapubic midline incision approximately 1 cm above the pubic symphysis was made to expose the bladder. A subcutaneous tunnel was created between the cranial and abdominal incisions, and the distal catheter was advanced through this tunnel. After saline instillation confirmed adequate bladder filling, the distal catheter was inserted into the bladder lumen and secured by careful closure of the bladder wall around the catheter.

Postoperatively, older children were advised to avoid prolonged urinary retention. All patients received routine perineal hygiene care and a 7-day course of an oral second-generation cephalosporin according to the institutional perioperative practice used during the study period. The present retrospective study was not designed to evaluate the efficacy of this antibiotic regimen in preventing urinary or shunt infection. Serum electrolytes and clinical signs of infection or shunt dysfunction were monitored closely.

## 2 Results

All 36 VVS procedures were technically completed without a major intraoperative complication documented in the available operative records. Follow-up ranged from 6 to 24 months, with a mean duration of 13.44 ± 8.21 months.

At the most recent available follow-up, radiological improvement of hydrocephalus was reported in 27 patients. Seven additional patients remained clinically stable without documented progression. Two patients discontinued treatment and were subsequently lost to follow-up.

Five of 36 patients (13.9%) developed postoperative intracranial infection. Four patients (11.1%) developed infection within 1 month after VVS placement, and all four had a previous history of bacterial meningitis. Three improved following antibiotic treatment, whereas one discontinued treatment. In one of these patients, the postoperative pathogen was reported to be the same as that identified during the preceding episode of purulent meningitis; the other postoperative infections involved organisms different from the preceding infectious episode. A fifth patient, who had originally undergone VVS because of recurrent abdominal ascites, developed an intracranial infection approximately 2 years after surgery. This patient responded clinically to antibiotic treatment but was subsequently lost to follow-up. Organism-specific microbiological findings and shunt management are summarized in Table 2 to the extent that they could be reliably reconstructed from the original records. Because a urinary source was not systematically investigated at each episode, these infections cannot be attributed to ascending urinary infection on the basis of the available data.

**Table 2:** Characteristics and outcomes of postoperative intracranial infections after ventriculovesical shunting.

Case	Original VVS clinical scenario	Time from VVS to infection	Previous CNS infection	Organism before VVS	Organism after VVS	Treatment	Shunt retained/revised/removed	Final outcome
1	Persistent CSF abnormality after bacterial meningitis	14D	Bacterial meningitis	Staphylococcus epidermidis	Staphylococcus epidermidis	Intravenous vancomycin	retained	improved
2	Persistent CSF abnormality after bacterial meningitis	8D	Bacterial meningitis	Streptococcus agalactiae	Klebsiella pneumoniae	Intravenous meropenem	retained	improved
3	Persistent CSF abnormality after bacterial meningitis	11D	Bacterial meningitis	Streptococcus pneumoniae	Staphylococcus epidermidis	Intravenous vancomycin	retained	improved
4	Persistent CSF abnormality after bacterial meningitis	18D	Bacterial meningitis	Staphylococcus epidermidis	Escherichia coli	Intravenous meropenem	retained	later lost to follow-up
5	Refractory CSF ascites	23M	None	None	Klebsiella pneumoniae	Intravenous meropenem	NR	later lost to follow-up

Three patients (8.3%) developed symptomatic hyponatremia after discharge, with serum sodium concentrations ranging from 118 to 127 mmol/L. They presented with symptoms including fatigue and somnolence and were readmitted for sodium supplementation. Symptoms and sodium levels improved after treatment, and no recurrence was documented while oral sodium supplementation was continued.

No clinically recognized shunt obstruction or significant overdrainage was documented among patients who remained under neurological follow-up.

Eleven patients (30.6%) subsequently underwent conversion to VP shunting after the clinical circumstances that had initially precluded peritoneal diversion had improved, and their VVS systems were removed. Of the remaining 25 patients, two were lost to follow-up. Among the 23 patients with available follow-up (6–24 months), the VVS remained *in situ* at the last documented follow-up, with no recorded shunt revision, replacement, removal, or conversion to another CSF diversion procedure. Formal longitudinal assessment of VVS function was not systematically available from the retrospective records; therefore, long-term shunt durability could not be determined. VVS was used at our institution primarily as a bridging or salvage option in patients for whom VP shunting was temporarily unsuitable.

Patients were followed clinically and by neuroimaging as clinically indicated for assessment of hydrocephalus and shunt function. Blood counts and serum electrolytes were obtained according to clinical need, particularly when symptoms suggestive of infection or electrolyte disturbance occurred.

Importantly, no standardized long-term urological surveillance protocol was implemented during the study period. Serial scheduled urinalysis or urine cultures were not routinely performed in asymptomatic patients, and renal/bladder ultrasonography, post-void residual measurement, urodynamic testing, and voiding cystourethrography for vesicoureteral reflux were not systematically undertaken during follow-up. Therefore, the study was not capable of reliably determining the incidence of asymptomatic bacteriuria, microbiologically confirmed urinary tract infection, catheter encrustation or bladder stones, bladder dysfunction, vesicoureteral reflux, or upper urinary tract consequences.

Bladder calculi were documented in three patients. The calculi were identified intraoperatively during subsequent procedures performed 6 months, 14 months, and 18 months after the preceding surgery, respectively, and were removed during the same procedure in all three patients. Because patients did not undergo systematic surveillance for bladder calculi, complete ascertainment of stone events could not be assured. Accordingly, these three cases are reported as documented events only, and an incidence was not calculated. During retrospective follow-up, some patients or caregivers reported symptoms considered compatible with urinary tract infection; however, these episodes were not verified by contemporaneous urinalysis or urine culture and were therefore not classified as confirmed UTIs. (Representative imaging findings are shown in Figures 1–3).

**Figure 1:**
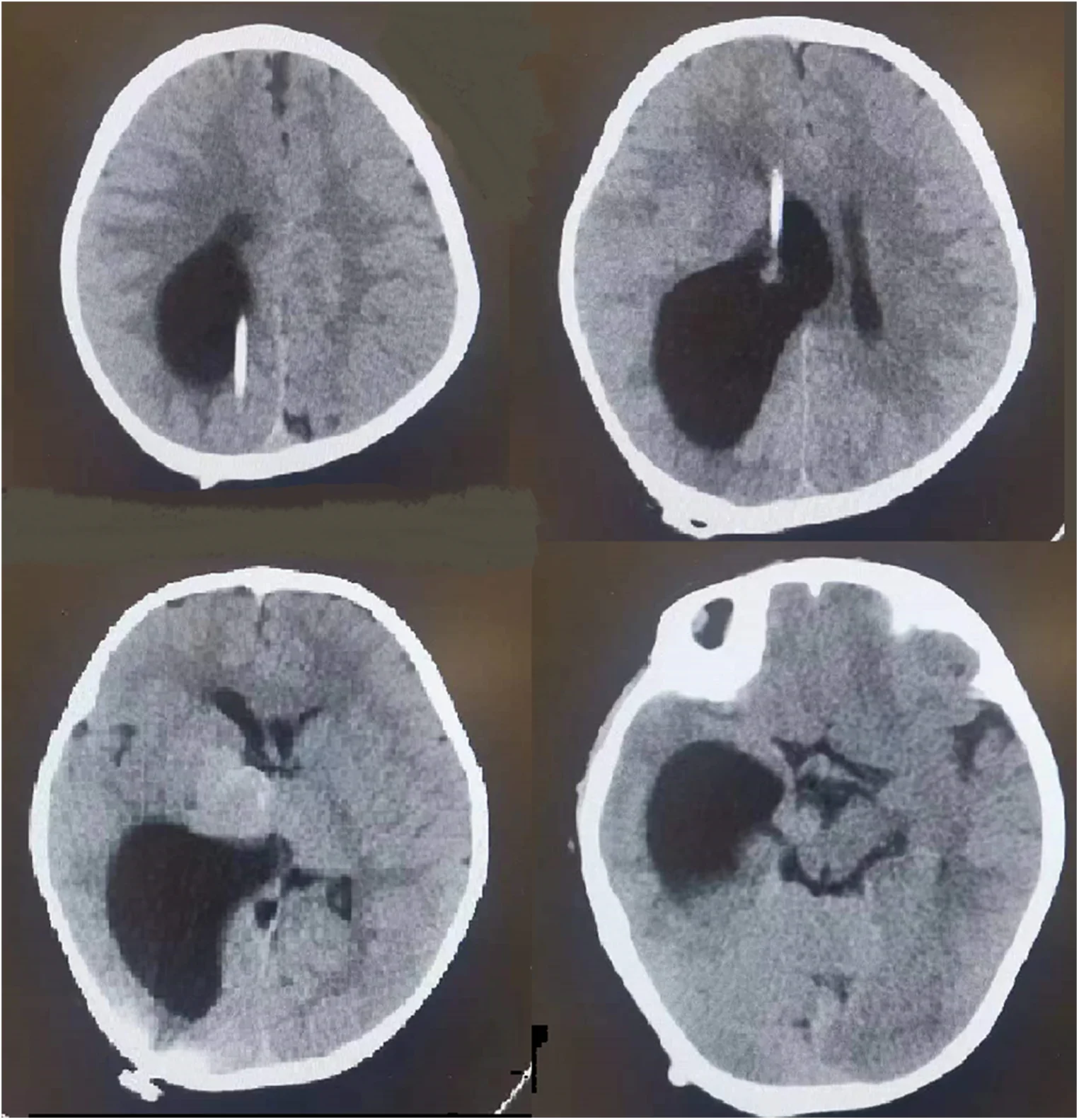
A 2-year-old male child with elevated cerebrospinal fluid protein levels following treatment for encephalitis. Preoperative CSF protein level was 2.6 g/L, with concomitant ventricular adhesions. Postoperative head CT performed on day 3 shows the clinical outcome.

**Figure 2:**
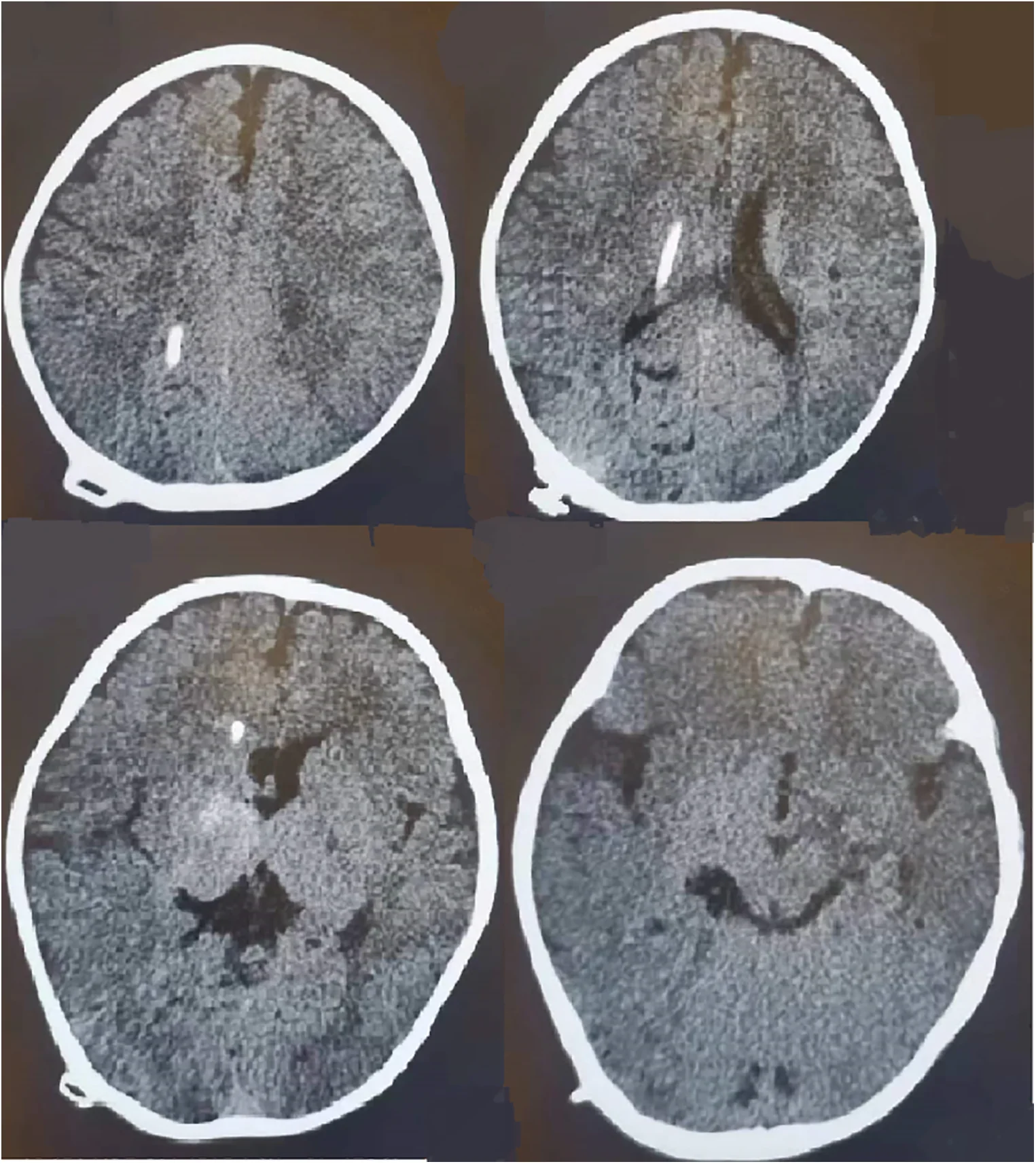
Postoperative head CT at 3 months following ventriculo-bladder shunting, showing significant improvement in hydrocephalus.

**Figure 3:**
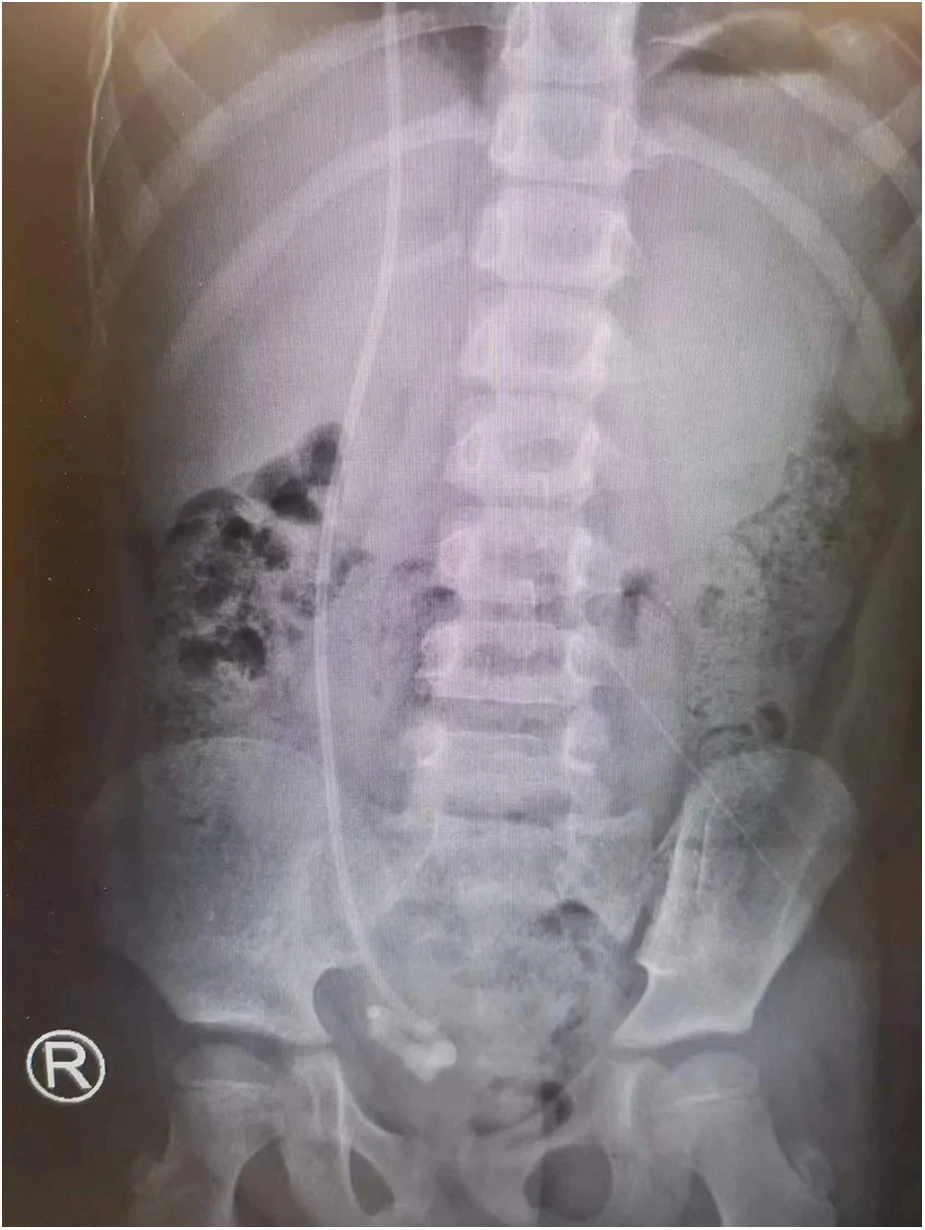
Postoperative upright abdominal x-ray showing the distal end of the shunt positioned in the pelvic region.

## 3 Discussion

VP shunting remains a standard treatment for pediatric hydrocephalus, but the peritoneum cannot always serve as an acceptable distal recipient site. The present series describes 36 children in whom our treating team selected VVS because peritoneal diversion was considered undesirable or not feasible at that point in the child's clinical course. The principal interpretation of our findings is descriptive: VVS provided internal CSF diversion during the available follow-up in a selected salvage population, and 11 children were subsequently converted to VP shunting when circumstances permitted. These observations do not demonstrate that VVS is superior to other nonperitoneal diversion strategies or establish evidence-based indications for its use.

Persistently elevated CSF protein requires particular clarification because it accounted for 17 of 36 patients in this series. The published relationship between CSF protein and shunt obstruction is inconsistent (9, 11). Earlier prospective and catheter-material studies found little evidence that elevated CSF protein at the time of shunt insertion independently increased shunt complications or that protein deposition alone was sufficient to explain catheter obstruction. More recent studies have suggested that persistent CSF protein abnormalities or higher protein concentrations may be associated with early valve occlusion or shorter shunt survival in selected populations, including disease-specific cohorts (12, 13). Consequently, no universally accepted CSF protein threshold can presently be used to define VP-shunt ineligibility (12, 14).

Equally importantly, VVS does not bypass the exposure of the ventricular catheter, valve, or proximal portion of the distal catheter to protein-rich CSF. Therefore, the fact that CSF constituents entering the bladder may subsequently be excreted in urine does not establish that VVS prevents protein- or cell-related shunt obstruction. Urinary elimination of CSF constituents formed part of our institutional rationale when this strategy was developed, but it remains a clinical hypothesis rather than a demonstrated mechanism of improved shunt patency. In the present study, the more defensible rationale for VVS in these patients is that it provided a nonperitoneal recipient site while persistent CSF abnormalities and other clinical circumstances made immediate peritoneal diversion less desirable in the judgment of the treating team.

VVS should also be considered in the context of other nonperitoneal strategies. EVD offers rapid and controllable CSF diversion, particularly during active infection or while awaiting definitive treatment^4^, but is generally a temporary strategy and carries risks including infection, catheter malfunction, and prolonged inpatient management. ETV avoids an implanted distal shunt and is an important option in anatomically appropriate obstructive hydrocephalus (15); however, its success is strongly dependent on age, hydrocephalus etiology, and ventricular anatomy. In secondary ETV series, younger age and post-inflammatory hydrocephalus have been associated with a greater risk of failure (5).

VA and VPl shunts also represent alternative options for non-peritoneal distal catheter placement. Contemporary pediatric multicenter data indicate that both sites can be used when the peritoneum is unavailable, although each introduces recipient-site-specific risks (16). VA shunting requires consideration of intravascular and cardiopulmonary complications as well as catheter length during growth (6); VPl shunting may be complicated by pleural effusion, particularly in younger children (7). A recent institutional series of non-VP shunts consisted predominantly of VA and VPl procedures, with VVS used only exceptionally (8), illustrating the uncommon salvage position of the bladder as a distal recipient site. Thus, our results should not be interpreted as establishing VVS as the preferred alternative when VP shunting cannot be performed. Rather, selection should be individualized according to the child's anatomy, infection status, previous procedures, age, and organ-specific risks.

The urinary tract represents the most important incompletely assessed safety domain in the present study. A catheter chronically residing in the bladder may theoretically or clinically be associated with infection, encrustation or stone formation, altered bladder function, vesicoureteral reflux, and upper urinary tract consequenc (17, 18). Bladder stones associated with ventriculovesical shunts have been reported previously. More recently, a 2026 pediatric VVS series emphasized urodynamic bladder-pressure assessment and structured urological management as important components of patient selection and follow-up (8).

Our retrospective series did not include such a standardized surveillance program. The reports of symptoms suggestive of UTI during follow-up were not supported by contemporaneous urinalysis or cultures and therefore cannot be counted as confirmed UTIs. Similarly, bladder calculi were identified incidentally during evaluation for subsequent surgery, not through systematic screening, so their incidence cannot be reliably estimated. Most importantly, VUR, bladder dysfunction, and pressure-related bladder abnormalities were not systematically tested, and no structured long-term surveillance of the upper urinary tract was performed. The study therefore cannot establish that these complications were absent or determine the long-term urological safety of VVS. This represents a major limitation and should be addressed prospectively in any future evaluation of the technique.

Postoperative intracranial infection was another clinically important complication. Five patients (13.9%) developed intracranial infection, including four within the first postoperative month. The early infections occurred in children with previous bacterial meningitis, and one patient had recurrence with the same organism as the earlier meningitic episode, whereas other infections involved different organisms. The mechanism of infection therefore cannot be assigned uniformly. Persistence or recurrence of a preceding infection, perioperative shunt infection, hematogenous infection, and ascending contamination from the urinary tract are possible considerations. Because paired urine cultures and systematic urinary investigations were not obtained, the present data cannot determine whether any infection originated from the bladder. Nevertheless, the anatomical communication established between the urinary bladder and ventricular system makes the possibility of ascending infection clinically important and reinforces the need for careful urological and infectious surveillance.

Hyponatremia developed in three patients. Although urinary loss after VVS is a possible mechanism, this retrospective study did not quantify urinary sodium excretion or perform a physiological study capable of establishing causation. We therefore interpret hyponatremia as an observed postoperative complication temporally associated with VVS rather than as proof of a specific sodium-loss mechanism. All three patients improved with sodium supplementation.

Several limitations substantially constrain interpretation. First, this was a retrospective, single-center study without a concurrent comparator, and patient selection reflected institutional practice rather than predefined evidence-based indications. Second, the sample was small and heterogeneous. Third, follow-up was relatively short (6–24 months), and two patients were lost to follow-up. Fourth, and most importantly for long-term safety, urinary outcomes were not prospectively or systematically collected: there was no standardized surveillance for UTI, bladder stones or catheter encrustation, VUR, bladder function, or upper urinary tract consequences. Therefore, the incidence of these events and long-term urological safety cannot be determined. Finally, the absence of a comparator precludes conclusions regarding superiority or relative efficacy compared with VA, VPl, ETV, EVD, or subsequent VP diversion.

Despite these limitations, the series contributes descriptive experience with an uncommon procedure in a difficult pediatric population. Its principal value is not to establish new contraindications to VP shunting, but to document how VVS was used as a selective salvage or bridging option in our institution and to identify safety issues that should be evaluated systematically in future studies.

## 4 Conclusions

Ventriculovesical shunting should not be regarded as a routine alternative to ventriculoperitoneal shunting or as a procedure with established CSF-protein-based indications. In this retrospective single-center experience, VVS provided internal CSF diversion during the available follow-up in a highly selected group of children in whom peritoneal diversion was considered unsuitable or undesirable at the time of treatment, and some patients were subsequently converted to VP shunting. These findings are therefore most appropriately interpreted as descriptive support for VVS as a potential salvage or bridging strategy.

The study does not establish that VVS reduces protein-related shunt obstruction, and elevated CSF protein alone should not be interpreted as a universally accepted contraindication to VP shunting. Moreover, long-term urological safety cannot be determined because urinary tract infection, catheter encrustation or stone formation, vesicoureteral reflux, bladder function, and upper urinary tract outcomes were not systematically evaluated. Prospective studies incorporating standardized neurological and urological assessment, longer follow-up, and comparison with other nonperitoneal diversion strategies are required before the indications, durability, and long-term safety of VVS can be defined.

## Data Availability

The original contributions presented in the study are included in the article/Supplementary Material, further inquiries can be directed to the corresponding author.
